# Reviews, Notices, &c.

**Published:** 1857-08

**Authors:** 


### REVIEWS, NOTICES, &c.

How to Write—How to Talk—How to Behave—How to Do Business.—Four volumes, thirty cents each, or the four for one dollar. By Fowler & Wells, 308 Broadway, New York. These are useful books, and well worthy of a place in the library of every young man and woman in the land.

"The Country Gentleman," Albany, N. Y., $2 a year, is a weekly publication on agricultural subjects, which merits a wide patronage. It is edited with industry and ability, and ought to be liberally sustained.

Littell's Living Age, weekly, Boston ; $6 a year, 8vo.—Dialogues on Divine Providence, No. 685—Vision of a Studious Man, No. 684—which ought to be read and studied by every married person living. Edgar Allan Poe, No. 686—Goldsmith, by Macaulay, No. 670—Charlotte Bronté and Sir John Franklin. No. 683. In fact, every No. of Littell has one or more rich articles of permanent value. Single Nos. sent, post-paid, for twelve cents.

Chicago Magazine; or, *The West as it is.* June No. has twelve illustrations—History of Chicago, Biographies, Poetry, Sketches, &c. $3 a year.

Blackwood, No. 81, opens with a novel by *Pisistratus Caxton*, What will He Do with It?—The Athelings—New Sea-side Studies—American Explorations—Scenes of Clerical Life. $3 a year.

Scalpel, for July ; $1 a year. Mechanically, the neatest and comeliest quarterly we receive ; and as to contents, without a second. Whatever Dr. Dixon chooses to write about, he will command readers. He writes rousingly, and always instructively. Even in defending indefensible positions, wholesome truths sparkle out with delighting profusion.

We commend to our subscribers, scattered throughout the wide West, that excellent Agricultural Weekly, *The Ohio Farmer*, published at Cleveland, O., at two dollars a year. American Agriculturist does not reach us.

We are indebted to our indefatigable City Inspector, G. W. Morton, Esq., for Mortality Reports for 1855, bound in muslin ; and also, to Mr. Geo. T. Falls, his Secretary, for an accurate and neatly-prepared abstract of items for 1857—from all of which, we are selecting some suggestive facts for a future number. Will the proper authorities hurry up the "Reports" for 1856.

The Messrs. Wood, of 389 Broadway, have issued the second edition, revised and improved, of *Brown's Grammar* of English Grammar, 1070 pages, 8vo, with a beautifully-executed engraving of the Author, lately deceased. It is sent, p. p., for Five Dollars, and is believed, by many of the first scholars in the land, to be the most complete and elaborate English Grammar ever published. It should be in the library of every professional man in the country, of every scholar, and of every teacher, who can at all afford it. We will notice it again.

Dinsmore's Railway Guide, 9 Spruce street, arranged monthly, gives every desired information as to all the lines of travel in the Union. An indispensable vade mecum, in the travelling season especially.

Sewing Machines, at $75 each, are for the rich only, and they do not want them, unless it be some notable matron, like *Jessie Fremont,* who has been learning how to use them. We are glad to learn that Watson has invented a sewing machine, for ten dollars, which a child can easily work. E. Sampson, of Ypsilanti, Michigan, supplies them for that region. Mr. Watson should make known his whereabouts.

